# RNA–Protein Reciprocity and the Limits of RBP Buffering

**DOI:** 10.1002/wrna.70065

**Published:** 2026-10-16

**Authors:** Epaminondas Doxakis

**Affiliations:** ^1^ Center of Basic Research Biomedical Research Foundation, Academy of Athens Athens Greece

**Keywords:** alternative polyadenylation, binding‐site competition, biomolecular condensates, post‐transcriptional regulation, RNA regulation, RNA‐binding proteins, RNA–protein stoichiometry

## Abstract

RNA‐binding proteins (RBPs) regulate RNA processing, localization, translation, and decay, yet similar perturbations can have different consequences across cells and tissues. RNA–protein reciprocity relates this variation to the balance between the binding sites presented by RNA and the protein available to regulate them. Regulation can remain stable while sufficient RBP is available, but become sensitive to small changes as that reserve narrows. The biological consequence depends on which transcripts lose regulation and how strongly the cell depends on their products. Examples from endogenous regulatory RNAs, repeat‐expansion diseases, viral infection, and cancer show that RNA‐dependent RBP redistribution can preserve cellular function or contribute to its failure. Binding affinity, partner proteins, autoregulation, and RNA‐dependent assembly shape these responses. This Perspective develops the framework and proposes experiments to connect changes in local RNA demand and RBP supply with target binding and biological output. The aim is to identify when a modest molecular perturbation has a disproportionate functional effect, and when restoring the RNA–protein balance can recover regulation.

## Graphical abstract

Effective transcript demand (D) reflects accessible binding sites weighted by affinity; usable RBP supply (S) is the protein available to bind them in the same compartment. The load ratio L = D/S describes their balance, which can influence regulatory reserve and target regulation.

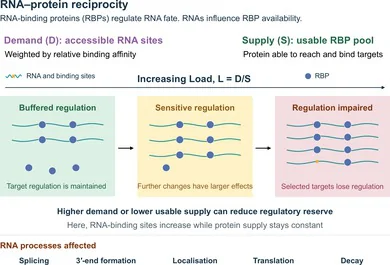

## 1 Introduction

The consequences of an RNA‐binding protein (RBP) perturbation depend on which transcripts require it in a particular cell. TDP‐43 illustrates this dependence. Its repression of cryptic exons was established in mouse embryonic stem cells, with loss of this repression also detected in amyotrophic lateral sclerosis and frontotemporal dementia (ALS‐FTD) tissue (Ling et al. 2015). Deletion of TDP‐43 in mouse neurons and myocytes affected largely different cryptic exons, showing that its RNA‐processing requirements differ between cell types (Jeong et al. 2017). In human neuronal models, loss of nuclear TDP‐43 permits cryptic exon inclusion in *UNC13A*, reducing the expression of a protein required for synaptic vesicle release. ALS‐FTD risk variants increase this splicing defect, linking a specific RNA‐processing vulnerability to disease susceptibility (Brown et al. 2022; Ma et al. 2022). Other heterogeneous nuclear ribonucleoproteins (hnRNPs) also modify *UNC13A* splicing, and TDP‐43 loss causes widespread alternative polyadenylation (APA) changes (Koike et al. 2023; Zeng et al. 2025). These findings raise a broader question. What determines how much disturbance an RNA programme can tolerate before cellular function is compromised?

Earlier frameworks moved the discussion towards a broader understanding of post‐transcriptional control. The RNA regulon concept positioned RBPs as coordinators of multi‐transcript programmes (He et al. 2023; Hentze et al. 2018; Keene 2007; Keene and Tenenbaum 2002; Van Nostrand et al. 2020). Competing endogenous RNA (ceRNA) models proposed that transcripts sharing microRNA sites could influence one another by competing for the same regulator (Denzler et al. 2014; Salmena et al. 2011). Quantitative studies showed that this depends on the relative amounts of regulator and target sites. In some systems, very large numbers of added sites were needed to release endogenous targets from repression. Susceptibility also differed between microRNA families according to their abundance and the affinities of their target sites (Bosson et al. 2014; Denzler et al. 2014, 2016). The key lesson for RBPs is that shared binding sites enable competition, while abundance and binding properties determine whether it affects regulation.

RNA–protein reciprocity considers both directions of regulation. RBPs control RNA fate, while RNAs influence the availability and activity of the proteins that bind them. Competition provides one route. If one RNA population binds more of a shared RBP, less protein may remain for other targets; reducing that population may release protein for them. These effects are local. An RBP can become limiting in the nucleus or a neuronal process even when its whole‐cell abundance remains high. The framework describes this balance using effective transcript demand (D), the accessible binding sites for a particular RBP, and usable supply (S), the protein able to bind those sites in the compartment studied (Figure 1; Box 1). Changes in RNA abundance or 3′UTR usage alter demand, whereas relocalization or loss of binding competence can reduce supply (Arimoto‐Matsuzaki et al. 2016; Guil et al. 2006; Hofmann et al. 2021; Kedersha et al. 2000; Pereira‐Castro and Moreira 2021; Tushev et al. 2018). Regulation is buffered when these changes leave sufficient protein available to maintain the relevant RNA programme. Near the limit of this reserve, a further small perturbation may produce a disproportionate change in RNA processing. If the affected transcripts sustain an essential function, the resulting phenotype may appear abruptly. The framework predicts that the same perturbation can be tolerated in one cell but disrupt function in another. Their reserve and dependence on the affected transcripts help determine the difference.

**FIGURE 1:**
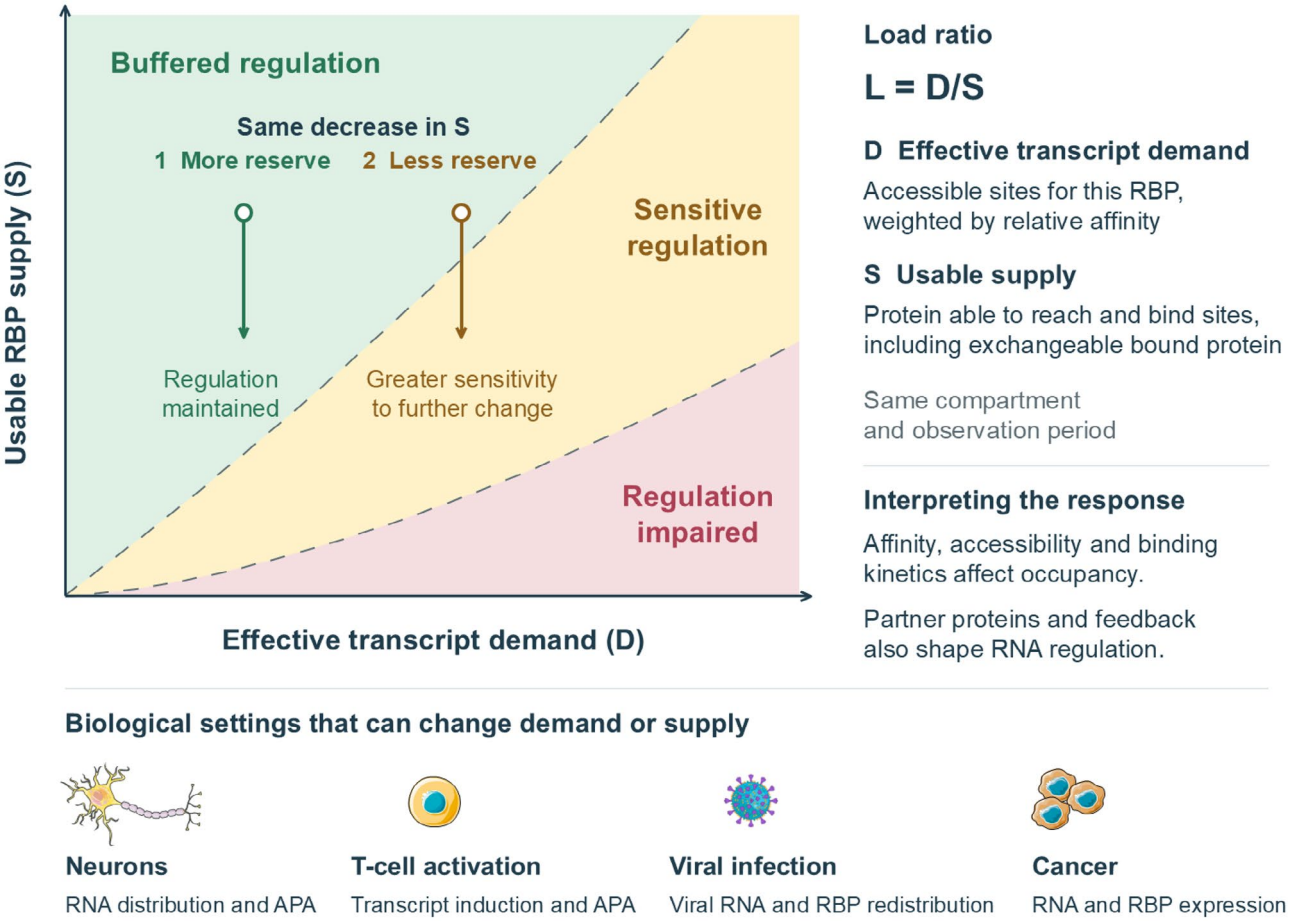
Transcript demand, usable RBP supply and regulatory reserve. The conceptual map relates effective transcript demand (D) to usable supply (S) for one RBP in a specified compartment and observation period. D summarizes accessible RNA binding sites, weighted by relative affinity. S includes protein able to reach and bind those sites, including reversibly bound protein that can exchange between targets. The load ratio L = D/S describes relative demand. Shaded regions distinguish buffered regulation, greater sensitivity to changes in D or S, and impaired regulation of the selected RNA programme. Equal downward arrows show the same reduction in S from starting states with different reserve. Regulation remains buffered in example 1, whereas example 2 enters the more sensitive range. The biological illustrations identify settings in which RNA abundance, alternative polyadenylation (APA), protein distribution, or RNA binding can change demand or supply. These settings are shown below the map without assigned positions. Boundaries are schematic, and no fixed L value defines a transition. Absolute concentrations, affinity, accessibility, and binding kinetics affect occupancy. Partner proteins and feedback also shape regulation. A steep regulatory response need not reflect an equally steep change in binding. Effects of RBP excess lie outside the range illustrated. Some elements in this image were obtained from Servier Medical Art and are used under CC BY 4.0.

Supply and demand act alongside other determinants of RBP function. RNA structure and binding affinity shape site occupancy, while partner proteins influence what binding achieves, as illustrated by *APP* splicing in Section 2. RNA decay removes sites, and autoregulation can replenish the protein pool. These processes help determine where a buffering boundary lies. Total RBP abundance does not reveal how much protein can reach and regulate a particular RNA population. Testing the framework therefore requires measurements of local RNA and protein abundance, RBP binding to selected targets, and the resulting regulation. Section 5 explains how controlled changes in demand or supply can connect these measurements and identify a contribution from competition. Neurons illustrate why this balance may matter for cellular function. Long 3′UTRs and RNA transport create distinct regulatory demands in the cell body, dendrites, and axons (Ciolli Mattioli et al. 2019; Doxakis et al. 2025; Hilgers et al. 2012; Tushev et al. 2018). During proliferation and immune activation, transcript induction adds RNA molecules while 3′UTR shortening removes some regulatory sites (Mayr and Bartel 2009; Sandberg et al. 2008). Viral infections introduce RNAs that engage host proteins and can redirect their functions (Manokaran et al. 2015). Sections 2, 4 examine how these changes affect RNA regulation and why the consequences range from adaptation to disease.

BOX 1 **Estimating transcript demand and usable RBP supply.**

Demand (D) and usable supply (S) refer to one RBP in the same compartment and over the same observation period. For quantitative comparisons, RNA sites and protein should be expressed in comparable units. Compartment volume is needed when converting amounts to concentrations.

D≈Σ (RNA abundance × accessible motif load × affinity weight).

Demand combines the number of RNA molecules with the accessible sites they carry. Each site is weighted by its binding strength relative to a reference site, using the same reference across conditions. D estimates the binding demand presented by RNA; it does not measure how many sites are actually occupied by the RBP. Large numbers of weak sites can also bind substantial protein.

S≈RBP amount in compartment × binding‐competent fraction.

Usable supply includes protein that can reach and bind the relevant RNAs during the period studied. Reversibly bound protein remains part of this pool if it can exchange between targets. The free fraction alone, therefore, underestimates supply. Conversely, a diffuse protein may have lost RNA‐binding competence, so diffuse abundance can overestimate it. Retention in an assembly reduces supply only to the extent that it prevents access to the relevant targets.

A competing RNA within the compartment increases D; protein bound to it remains part of S if it can exchange with other targets. Redistribution of the RBP out of that compartment reduces local S.

L = D/S describes effective RNA demand relative to usable RBP supply. A higher L indicates more demand per unit of usable protein, but does not by itself establish that regulation has failed. The same ratio can correspond to different absolute concentrations and levels of target binding. Regulatory failure also depends on which targets lose binding and how much binding their regulation requires. The sensitive range must instead be identified by measuring when changes in demand or supply begin to produce larger changes in target regulation.

## 2 Conditional Allocation

When usable RBP supply falls or RNA demand rises, transcripts sharing that protein may compete more strongly for binding. This redistribution is termed conditional allocation here (Figure 2). The effects need not be uniform across an RNA programme. Graded induction of MBNL1 in human HEK293 cells showed that different exon choices respond over different protein concentration ranges. Some changed at low MBNL1 levels, whereas others required higher protein levels. The *MBNL1* and *MBNL2* autoregulatory exons changed sharply over a narrow range, while most other events changed more gradually (Wagner et al. 2016). In RBFOX experiments, increasing protein concentration brought additional exons with intermediate‐affinity motifs under regulation (Begg et al. 2020). These titrations establish target‐specific sensitivity to protein concentration. Demonstrating competition additionally requires showing that changing one RNA population alters RBP binding and regulation at other targets. The biological impact will depend on the functions performed by that sensitive subset.

**FIGURE 2:**
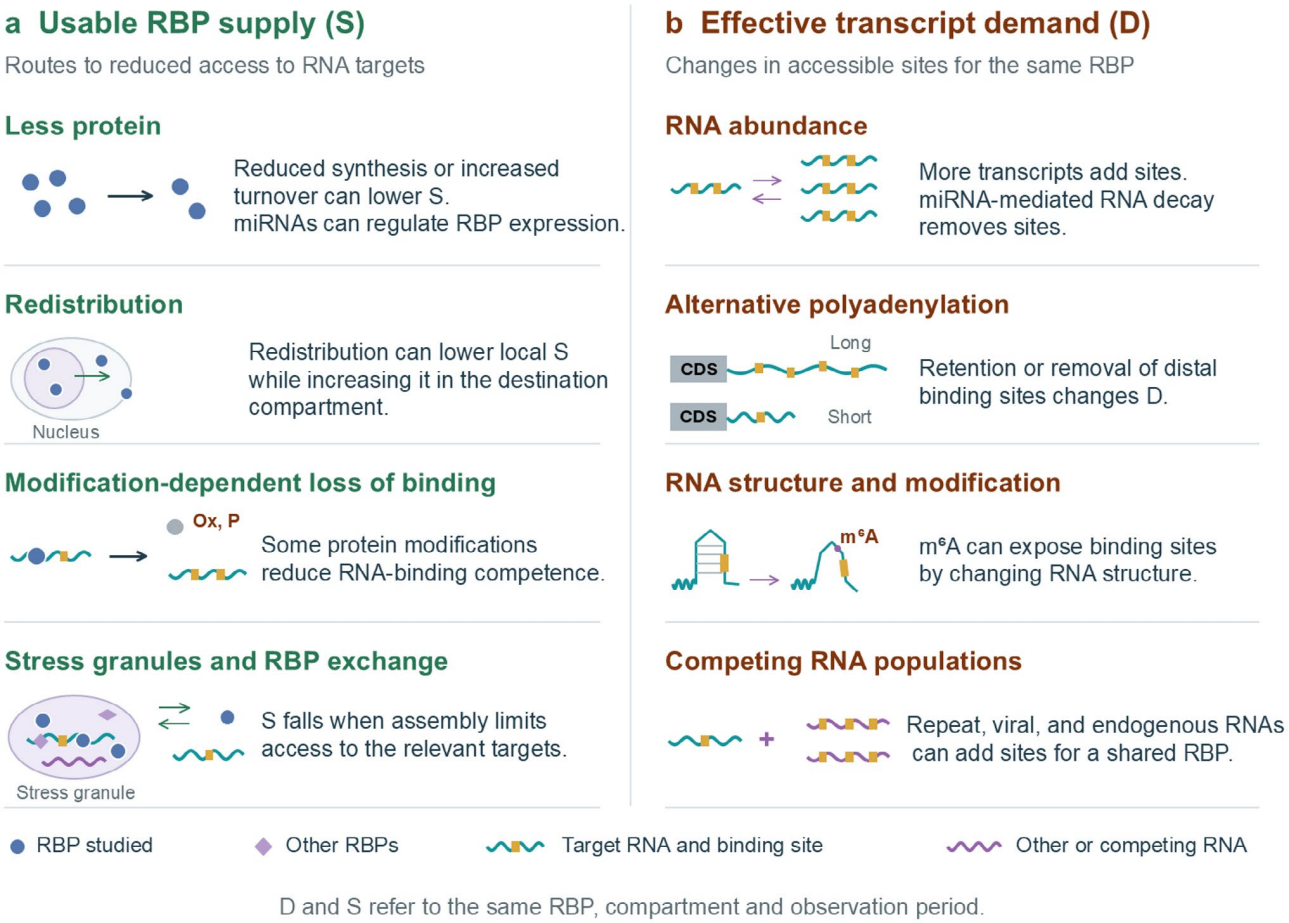
Mechanisms that change usable RBP supply and effective transcript demand. (a) Usable supply (S) can fall through reduced protein abundance, redistribution out of the compartment studied, modification‐dependent loss of RNA‐binding competence, or assembly that restricts access to relevant targets. MicroRNAs can regulate RBP expression. Redistribution can increase supply in the destination compartment. Ox and P indicate oxidation and phosphorylation, respectively. For example, hydrogen peroxide‐induced oxidation of TIA1 reduces its association with target mRNAs in human U2OS cells (Arimoto‐Matsuzaki et al. 2016). The stress granule contains the RBP studied, other proteins, and several RNAs. Exchange arrows indicate reversible movement between assembled and dispersed states. Recruitment into a granule does not, by itself, cause a loss of usable supply. (b) Effective demand (D) changes with RNA abundance, alternative polyadenylation, motif accessibility, and additional competing RNAs. MicroRNA‐mediated RNA decay removes binding sites, whereas RNA induction adds sites. The RNA‐structure example refers to N^6^‐methyladenosine (m^6^A)‐dependent exposure of hnRNPC‐binding sites in human cells (Liu et al. 2015). These effects depend on the RBP and RNA sequence. S and D refer to the same RBP, compartment, and observation period. When competing sites are included in D, their exchangeable bound protein remains within S.

*APP* splicing illustrates how partner proteins shape an RBP's regulatory effect. Neuronal ELAVL/Hu proteins (ELAVL2/3/4) promote skipping of *APP* exons 7 and 8, producing the neuron‐enriched APP695 isoform. They reduce binding of the splicing factor U2AF65, and concurrent AUF1 binding facilitates this effect (Fragkouli et al. 2017). The response therefore depends on the proteins assembled at the transcript as well as on ELAVL availability. At AU‐rich elements (AREs) in cytokine and growth‐control transcripts, HuR and AUF1 can bind shared targets concurrently or compete. Their associations also differ between the nucleus and the cytoplasm (David et al. 2007; Lal et al. 2004; Zou et al. 2010). Competition between proteins at one RNA can consequently modify the response to competition between RNAs for an RBP pool.

RNA processing determines which binding sites are available to RBPs. APA changes 3′UTR length through alternative cleavage‐site selection, which is influenced by RNA polymerase II elongation and recruitment of processing factors (Mitschka and Mayr 2022). In fission yeast, slower elongation favors proximal polyadenylation sites and shorter 3′UTRs (Yague‐Sanz et al. 2020). U1 snRNP suppresses premature cleavage and polyadenylation, preserving downstream RNA sequence (Kaida et al. 2010). Partial loss of CFIm25 alters hippocampal APA and causes cortical hyperexcitability and learning deficits in mice. In human stem cell‐derived neurons, its reduction causes widespread 3′UTR shortening (Alcott et al. 2020). Neuronal ELAV promotes distal site selection and 3′UTR extension in *Drosophila* (Hilgers et al. 2012). These examples connect RNA architecture with neuronal function. The reciprocity framework adds a further prediction. Gaining or losing sites on one RNA population may alter the protein available to regulate other transcripts.

Stress can change which RNA populations an RBP can reach. In sorbitol‐stressed mammalian cells, hnRNPA1 phosphorylation weakens its interaction with the nuclear import receptor transportin‐1 (Trn1), promoting cytoplasmic accumulation and recruitment to stress granules (Allemand et al. 2005; Guil et al. 2006). After heat shock in HeLa cells, HuR associates more strongly with the export ligands pp32 and APRIL, and its nuclear export becomes CRM1‐dependent (Gallouzi et al. 2001). This redistribution can reduce the protein available to nuclear targets while increasing its abundance in the cytoplasm. Hydrogen peroxide can instead reduce RNA‐binding competence. In human U2OS cells, it oxidizes TIA1, decreasing TIA1 association with target mRNAs and impairing stress‐granule formation during thapsigargin‐induced endoplasmic reticulum stress. Combined exposure to hydrogen peroxide and thapsigargin increases apoptosis (Arimoto‐Matsuzaki et al. 2016). Here, loss of usable supply reflects impaired binding, with consequences for the cellular stress response.

IRP1 provides a physiological example of a switch in RNA‐binding competence. Assembly of a [4Fe–4S] cluster converts IRP1 to cytosolic aconitase and inactivates RNA binding. Mutagenesis implicated three of its four active‐site arginines in RNA binding (Philpott et al. 1994). Loss of the cluster under iron deficiency restores high‐affinity binding to iron‐responsive elements (IREs), allowing IRP1 to regulate target mRNA stability and translation (Wilkinson and Pantopoulos 2014). Iron availability can therefore change the functional RNA‐binding pool without changing total IRP1 abundance.

Restoring RBP supply can recover regulation, as shown by MBNL1 supplementation in a mouse repeat‐RNA model (Kanadia et al. 2006). Excess TDP‐43 can, however, promote the skipping of normally included exons. In TDP‐43 transgenic mice, total spinal‐cord TDP‐43 at approximately 1.5‐fold the control level was accompanied by constitutive exon skipping. A largely different set of exons was skipped in human induced neurons with total TDP‐43 at approximately 1.6‐fold the control level (Carmen‐Orozco et al. 2024). In inducible QBI‐293 cells, a nuclear‐localization mutant caused less constitutive exon skipping than wild‐type TDP‐43 despite a larger increase in total protein, consistent with exon skipping depending on the amount of TDP‐43 in the nucleus. Short UG motifs near the affected exons are consistent with additional binding at suboptimal sites. Both shortage and excess can therefore disturb splicing, although the excess‐associated events were not associated with disease status in the human samples examined (Carmen‐Orozco et al. 2024). For intervention, the goal is to restore the protein's functional range.

Autoregulation can limit the effect of a dosage change. *Tardbp* heterozygous mice retained near‐normal TDP‐43 protein despite reduced gene dosage (Sephton et al. 2010), consistent with feedback control of TDP‐43 expression (Ayala et al. 2011). This compensation explains why gene dosage need not translate directly into a change in usable protein. It also suggests a way to sustain buffering as RNA demand changes, a possibility examined in Section 5.4.

## 3 Riboregulation and RNA‐Side Modifiers

RNA regulation extends to the proteins that bind RNA. The broader principle of riboregulation includes RNA‐dependent changes in protein activity, localization, and assembly (Hentze et al. 2025). These effects fall under reciprocity because RNA can modify the regulator itself, as well as its access to other transcripts. Additional RNA can therefore alter RBP activity as well as compete for binding.

The cytoplasmic long non‐coding RNA *NORAD* provides an endogenous example. In human HCT116 cells, approximately 500–1000 *NORAD* molecules, each with 15 conserved PUMILIO response elements, provide an estimated 7500–15,000 potential sites. Quantitative immunoblotting estimated approximately 15,000 PUM1 and 2000 PUM2 molecules per cell (Lee et al. 2016). These comparable site and protein numbers support substantial sequestration capacity, although they do not measure the fraction of PUMILIO bound to *NORAD*. PAR‐CLIP confirmed PUMILIO binding to *NORAD* and identified other PUM2 targets. *NORAD* loss increases repression of PUMILIO targets and chromosomal instability, while reducing PUMILIO activity suppresses the phenotype. Subsequent work showed that NORAD‐induced condensates recruit additional PUMILIO through protein–protein interactions. This increases sequestration beyond RNA‐site binding alone and is required for genome stability in the tested cells (Elguindy and Mendell 2021). *NORAD* therefore helps restrain PUMILIO activity through multiple binding sites and protein assembly, protecting other transcripts from excessive repression.

RNA can also protect proteins from excessive assembly. In reconstituted systems, low RNA‐to‐protein ratios promote phase separation of prion‐like RBPs, whereas high ratios suppress it. Reducing nuclear RNA in cells promotes excessive phase separation (Maharana et al. 2018). RNA thus contributes to the physical buffering of the protein pool. Increasing RNA can preserve protein solubility while also increasing competition for binding; reducing RNA can relieve competition yet favor excessive assembly. The net effect on regulation depends on whether the protein remains accessible to its functional targets. Condensate formation does not by itself show that RNA regulation has been impaired. Assemblies can bring an RBP together with its targets or separate it from RNAs elsewhere in the cell (Putnam et al. 2023). Processing bodies, for example, contain poorly translated mRNAs and decay factors, but their abundance alone does not measure the rate of mRNA decay (Franks and Lykke‐Andersen 2008). The relevant tests are whether the RBP still binds its target and whether the target's processing, stability, or translation changes.

Expanded repeat RNA can retain RBPs and reduce their availability for normal targets. In muscle from patients with myotonic dystrophy, muscleblind proteins accumulate in nuclear foci containing expanded repeat RNA (Mankodi et al. 2001). In a mouse model expressing expanded CUG RNA, raising MBNL1 in skeletal muscle corrects several splicing defects and reverses myotonia (Kanadia et al. 2006). This rescue connects reduced RBP availability with a physiological defect. *C9orf72*‐associated ALS and FTD show how repeat expansions can also disrupt protein localization (Mizielinska et al. 2025). Models expressing the GGGGCC expansion show disrupted nucleocytoplasmic transport (Freibaum et al. 2015). Repeat‐derived proteins can contribute independently of the expanded RNA. In HeLa cells, dipeptide repeat proteins expressed from alternative‐codon constructs cause nuclear‐envelope abnormalities and mislocalisation of Ran and RanGAP, two regulators of nucleocytoplasmic transport. Cytoplasmic TDP‐43 accumulation occurs in cells with these transport abnormalities (Ryan et al. 2022). Such transport defects could reduce TDP‐43 access to nuclear targets.

MicroRNAs can change RBP supply by controlling protein expression. In mouse neuronal differentiation models, miR‐124 represses PTBP1 and promotes neuron‐specific splicing. PTBP1, in turn, can restrain miR‐124 biogenesis by binding pri‐miR‐124‐1. Its decline therefore reinforces the switch towards neuronal splicing (Makeyev et al. 2007; Yeom et al. 2018). RBPs can also influence microRNA action at a shared target. In HeLa cells, HuR binds the *MYC* 3′UTR beside a let‐7 site and promotes repression by the let‐7‐loaded RNA‐induced silencing complex (RISC) (Kim et al. 2009). Target‐directed microRNA degradation provides another route for RNA to change its regulator. Highly complementary targets can trigger degradation of bound microRNAs, reducing their availability for other transcripts (de la Mata et al. 2015; Shi et al. 2020). An RNA can therefore change the size of a regulatory pool as well as compete for binding to it.

RNA modifications can change which proteins bind a transcript and where those interactions occur. In human cells, N^6^‐methyladenosine (m^6^A) alters RNA structure to expose U‐tracts, increasing hnRNPC binding and affecting splicing (Liu et al. 2015). In the mouse hippocampus, the m^6^A reader YTHDF1 promotes translation of marked transcripts after neuronal stimulation; its loss impairs synaptic plasticity and learning (Shi et al. 2018). In HeLa cells, 5‐methylcytosine (m^5^C) deposited by NSUN2 and recognized by ALYREF promotes nuclear export (Yang et al. 2017). Modification can therefore change both an RNA's interaction with its regulators and the biological response that follows.

## 4 Reciprocity Across Biology

Neurons must sustain RNA regulation across a cell body and often extensive dendritic and axonal processes. Long neuronal 3′UTRs contain regulatory sites for RBPs and microRNAs, and alternative 3′UTR use influences transcript localization and stability (Ciolli Mattioli et al. 2019; Hilgers et al. 2012; Miura et al. 2013; Paschou et al. 2012; Tushev et al. 2018). RNA transport distributes these requirements between compartments (Baumann et al. 2022; Eom et al. 2003). The framework predicts that a neuronal compartment with little spare RBP capacity will be sensitive to a further increase in local RNA demand or loss of usable protein. Stress‐dependent redistribution and changes in granule assembly or disassembly provide routes by which that reserve could narrow (Garg et al. 2025; Goswami et al. 2023; Kedersha et al. 2000; Wolozin and Ivanov 2019). TDP‐43 illustrates the functional consequences of reduced RBP availability. Its depletion in the adult mouse brain preferentially reduces many transcripts with long introns, including genes involved in neuronal activity (Polymenidou et al. 2011). This nuclear processing requirement is distinct from the regulation of long 3′UTRs in neuronal processes. In human neuronal models, TDP‐43 binding to a GU‐rich region of *STMN2* pre‐mRNA suppresses cryptic splicing and premature polyadenylation through steric inhibition. Correcting that defect restores stathmin‐2 expression, axonal regeneration, and lysosome trafficking (Baughn et al. 2023; Figure 3a). These results show how failure to regulate a particular RNA can impair functions required to maintain an axon. The reciprocity hypothesis is that motor neurons may reach such functional limits after a smaller change in usable TDP‐43 than more resistant cells. Their vulnerability would reflect both the regulation of essential transcripts and their dependence on those transcripts to maintain long axons. Comparing these response ranges between cell types would test the proposed link between regulatory reserve and selective vulnerability.

**FIGURE 3:**
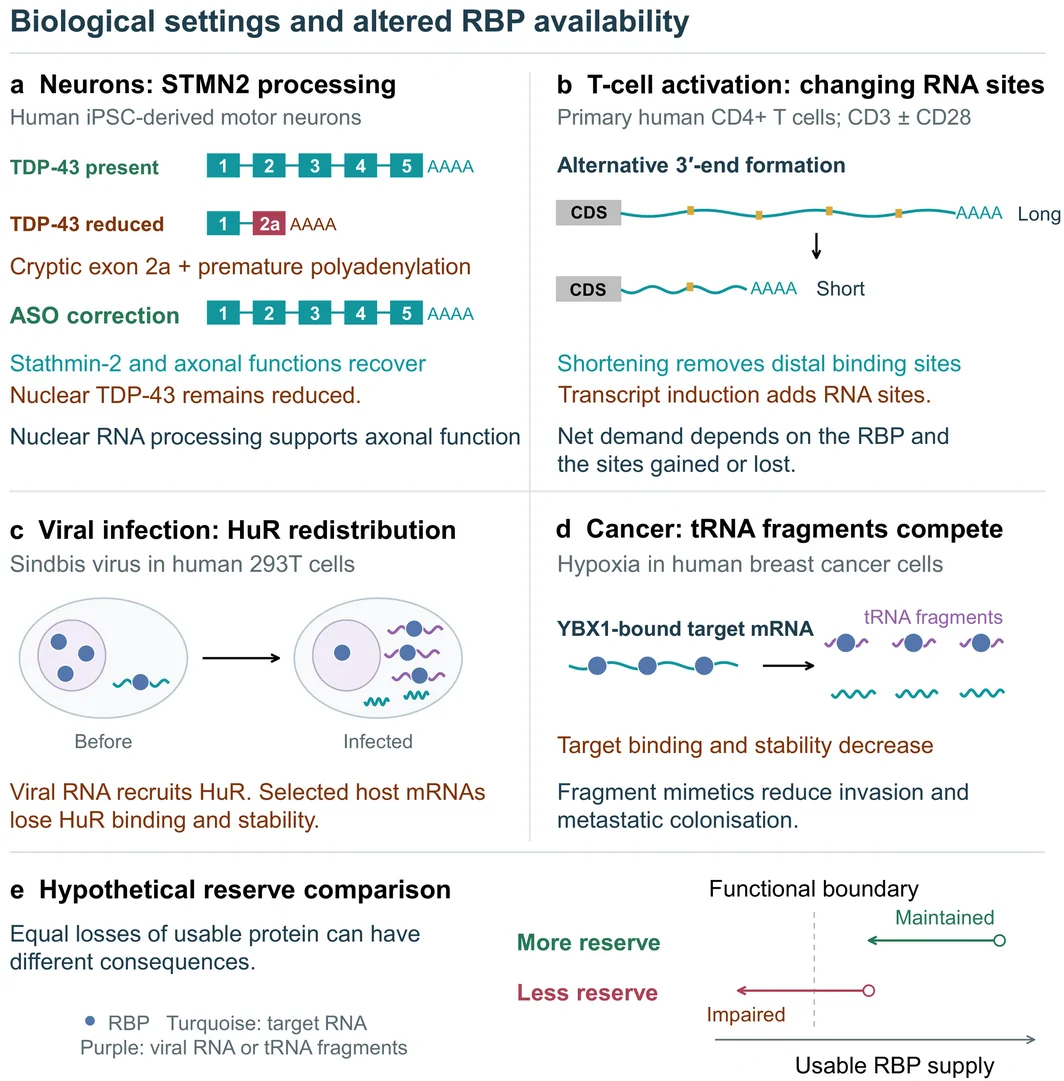
Biological settings, RNA regulation and the reserve hypothesis. Four biological settings illustrate changes in RNA demand, RNA‐binding protein (RBP) availability and target regulation. (a) TDP‐43‐dependent processing of *STMN2* pre‐mRNA links nuclear RNA regulation to axonal function. In human induced pluripotent stem cell (iPSC)‐derived motor neurons, TDP‐43 reduction permits splicing of *STMN2* exon 1 to cryptic exon 2a and premature polyadenylation. Premature termination excludes downstream exons and reduces full‐length stathmin‐2 expression. Splicing‐correcting antisense oligonucleotides (ASOs) restore stathmin‐2 and improve axonal regeneration and lysosome trafficking despite persistent TDP‐43 deficiency (Baughn et al. 2023). (b) Activation of primary human CD4+ T cells through CD3, with or without CD28 costimulation, changes alternative 3′UTR use in a time‐ and stimulus‐dependent manner (Blake et al. 2024). The illustrated shortening removes distal sites. Transcript induction and site loss can affect demand in opposite directions. (c) In Sindbis‐infected human 293T cells, HuR redistributes from the nucleus to the cytoplasm. Selected host transcripts lose HuR association and become less stable (Barnhart et al. 2013). (d) In human breast cancer cells, hypoxia induces tRNA‐derived fragments that bind YBX1 and displace target mRNAs, reducing their stability. Fragment mimetics reduce invasion in culture and metastatic colonization in mice (Goodarzi et al. 2015). (e) The reserve comparison shows a separate prediction: Equal reductions in usable protein can preserve regulation at greater starting reserve but impair it near a functional boundary. Starting positions and the boundary are hypothetical.

T‐cell activation changes RNA abundance and binding‐site content together. Activation‐associated APA tends towards 3′UTR shortening, with its extent depending on the stimulus and time after activation (Figure 3b) (Blake et al. 2024; Sandberg et al. 2008). Transcript induction adds molecules, whereas shortening removes distal sites, so demand on a particular RBP can move in either direction. For decay‐promoting RBPs, an increase in demand could prolong the lifetime of inflammatory transcripts if usable protein becomes limiting. ZFP36/TTP‐family proteins provide a biological setting for this prediction. TTP promotes *Tnf* mRNA decay in mouse macrophages, and ZFP36‐family proteins restrain activation of mouse T cells (Carballo et al. 1998; Moore et al. 2018). The framework predicts that transient competition among newly induced targets could amplify an inflammatory response, while replenishment of the regulator could help terminate it. Other regulators act at different stages. Roquin and Regnase‐1 recognize shared stem‐loop elements but regulate transcripts through routes linked to translation and the signaling state (Akaki et al. 2021; Behrens et al. 2021; Mino et al. 2015). An activation time course should therefore follow the specific regulator whose contribution is being tested.

Sindbis virus provides direct evidence that an added RNA population can redirect a host RBP. In infected human 293T cells, HuR accumulates in the cytoplasm and loses association with selected host mRNAs, whose half‐lives shorten two‐ to four‐fold (Figure 3c) (Barnhart et al. 2013). The affected messages include *DDX58*, which encodes the antiviral RNA sensor RIG‐I. Transfection of the viral 3′UTR reproduces HuR redistribution and selective transcript destabilization without viral proteins. Nuclear splicing and polyadenylation are also altered. Thus, cytoplasmic RNA can disrupt regulation in both compartments by recruiting a protein that normally shuttles between them.

Cancer‐associated RNA changes can likewise alter a regulatory programme. Shortening selected 3′UTRs can increase oncogene expression by removing regulatory sequence (Mayr and Bartel 2009). HuR abundance and localization are frequently altered in cancer (Schultz et al. 2020). Glutaminase converts glutamine to glutamate, which can be converted to α‐ketoglutarate to replenish the mitochondrial tricarboxylic acid cycle. The glutaminase C (GAC) isoform has higher catalytic activity than kidney‐type glutaminase (KGA) in phosphate‐activated biochemical assays (Cassago et al. 2012). In human breast cancer cells, HuR binds intron 14 of *GLS* pre‐mRNA and favors KGA expression. HuR depletion shifts the isoform balance towards GAC, accompanied by increased glutaminase activity and greater dependence on glutamine for mitochondrial respiration. It also increases sensitivity to glutaminase inhibition (Adamoski et al. 2024). Hypoxia and treatment‐associated stress also influence stress‐granule assembly and cancer‐cell adaptation (Jia et al. 2024; Redding and Grabocka 2023). A direct example of competition involves hypoxia‐induced tRNA‐derived fragments in human breast cancer cells. Fragments from tRNA‐Glu, ‐Asp, ‐Gly, and ‐Tyr bind YBX1, displacing oncogenic mRNAs and reducing their stability (Goodarzi et al. 2015). Antisense inhibition of the fragments increased invasion, whereas synthetic fragment mimetics reduced invasion and metastatic colonization. Highly metastatic derivatives showed weaker fragment induction. Here, endogenous RNAs compete for an RBP in a way that restrains malignant behavior, illustrating that competition can have a protective effect (Figure 3d).

The strongest examples connect RNA‐driven changes in RBP availability to substantial biological effects, including chromosome instability after *NORAD* loss, myotonia in repeat‐RNA models, and reduced invasion after YBX1 displacement. They establish that reciprocity can be influential, but do not yet show how often it limits regulation across RBPs and cell types. It is most likely to matter when a changing RNA population engages a substantial part of the local protein pool. High RNA abundance, clustered binding‐sites, or strong local accumulation can create this condition.

Sensitivity also depends on the starting state and the rate of change (Figure 3e). A cell already close to a functional boundary may respond to a modest perturbation, whereas a cell with more reserve may tolerate it. Autoregulation can replenish RBP supply (Muller‐McNicoll et al. 2019), and RNA‐dependent assembly can alter availability (Maharana et al. 2018). The framework therefore predicts that a gradual rise in demand may allow compensation, while an abrupt rise can produce a transient failure of regulation. This gives a testable explanation for sudden dysfunction after an apparently modest change.

## 5 Testing RNA–Protein Reciprocity

For RNA competition, the decisive question is whether increasing one RNA population reduces the protein available to regulate another. This can be tested by independently varying a competing RNA and its shared RBP, while keeping the target sequence unchanged. The competitor should carry validated binding sites, with a matched RNA lacking functional sites as the control. Both RNAs should be compared at similar abundance in the compartment studied. Competition predicts increased RBP binding to the competitor as its abundance rises, with reduced binding to the target followed by altered target regulation. Increasing RBP supply should maintain regulation at higher competitor levels. Early measurements help distinguish these effects from secondary changes in target RNA abundance, RBP abundance or localization, and stress signaling.

RNA fluorescence in situ hybridization combined with RBP immunofluorescence can establish the distributions of competitor, target, and protein. For short synthetic competitor RNAs, a biotin‐labeled version can be visualized with fluorescent streptavidin and used to recover bound proteins (Zhang et al. 2024). Cross‐linking and immunoprecipitation (CLIP) can compare RBP binding across conditions, accounting for differences in input RNA abundance and protein recovery (Xiang et al. 2024). Colocalisation CLIP can distinguish RBP interactions in the nucleus, cytosol, and arsenite‐induced stress granules (Yi et al. 2024). RBPscan uses RNA editing to compare binding across an RBP dose range. The RBP is fused to the catalytic domain of adenosine deaminase acting on RNA (ADAR), which introduces adenosine‐to‐inosine editing into an engineered recorder RNA carrying the tested binding site (Kretov et al. 2026).

### 5.1 Nuclear TDP‐43 and Cryptic Exon Repression

A direct test can start with a chemically stabilized (GU)_16_ RNA oligonucleotide described previously (Zhang et al. 2024). The corresponding (CA)_16_ oligonucleotide provides a sequence control, alongside mock transfection. Because (CA)_16_ can itself alter TDP‐43 localization, both controls are needed. Clip34nt, which binds TDP‐43 but has smaller effects on cryptic exon repression, provides an additional mechanistic comparator (Zhang et al. 2024). In human HeLa cells, (GU)_16_ impaired TDP‐43‐dependent cryptic exon repression in *EPB41L4A* and *ARHGAP32* without reducing steady‐state nuclear TDP‐43 localization.

Increasing (GU)_16_ doses can be tested at endogenous TDP‐43 levels and after a modest increase using a wild‐type TDP‐43 expression construct. Nuclear protein abundance and oligonucleotide accumulation should be measured in each condition. An early time course beginning before 6 h should compare TDP‐43 association with the oligonucleotide and endogenous target pre‐mRNAs alongside RT‐qPCR for cryptic and canonical splice junctions. This tests whether reduced target association precedes cryptic exon inclusion (Zhang et al. 2024). Pull‐down of biotinylated (GU)_16_ can measure TDP‐43 binding to the competitor, while cross‐linked TDP‐43 immunoprecipitation can measure its association with endogenous target pre‐mRNAs. Competition predicts increased oligonucleotide binding, reduced target association, and increased cryptic exon inclusion. If competition contributes to the splicing defect, additional TDP‐43 should reduce cryptic exon inclusion at the same measured nuclear oligonucleotide abundance (Figure 4).

**FIGURE 4:**
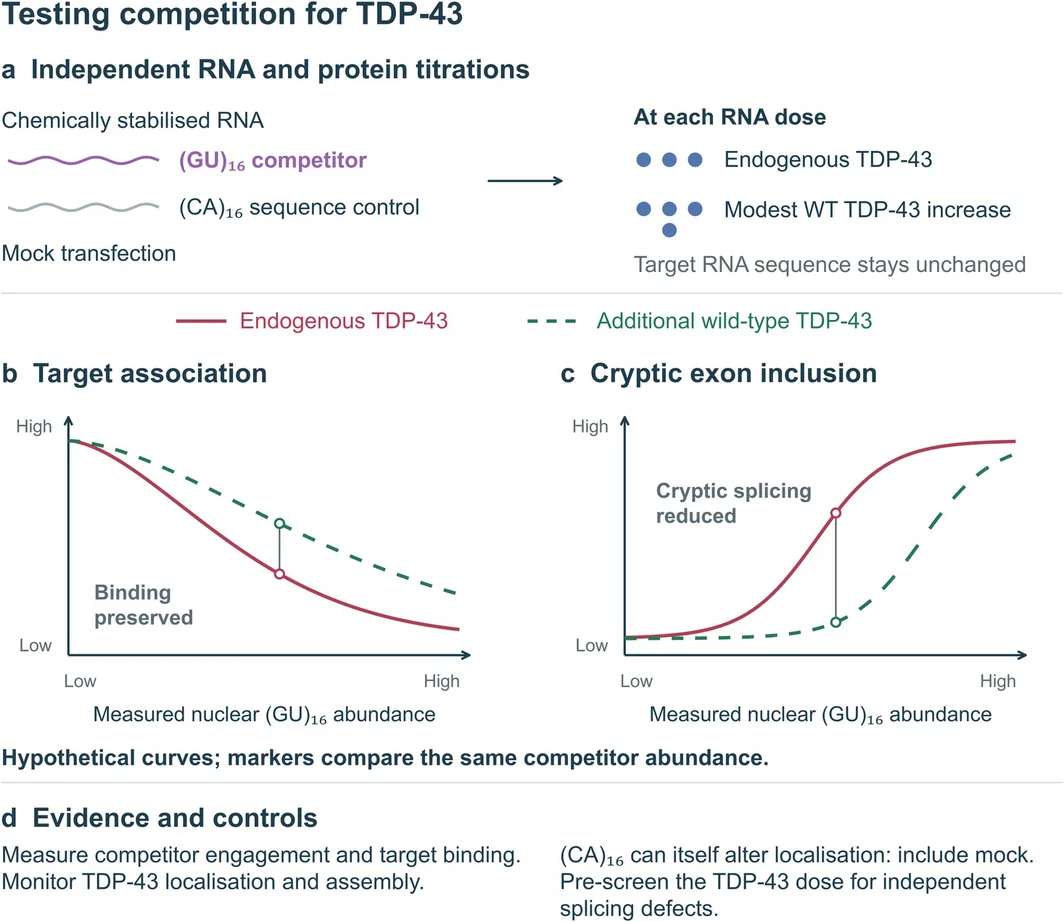
Testing RNA competition by varying competitor RNA and TDP‐43 independently. (a) A chemically stabilized (GU)_16_ RNA is titrated at endogenous and modestly increased wild‐type TDP‐43 levels, with (CA)_16_ and mock‐transfection controls. The reagent and cryptic‐splicing readouts follow published experiments in HeLa cells (Zhang et al. 2024); the paired titration and rescue are proposed extensions. (b, c) Hypothetical curves show target‐associated TDP‐43 and cryptic exon inclusion against measured nuclear competitor abundance. Added functional TDP‐43 is predicted to preserve target binding and reduce cryptic splicing. Open circles and the connecting vertical lines compare the two protein conditions at the same nuclear competitor abundance in each plot. Curve steepness describes sensitivity. (d) Evidence for competition also requires competitor engagement, binding‐site dependence, and protein rescue, with localization and assembly monitored. Because (CA)_16_ can itself alter TDP‐43 localization, mock‐transfection controls are also required. The supplementation dose must first be shown to leave both cryptic exon inclusion and constitutive exon skipping unchanged in mock‐transfected and control‐oligonucleotide cells. The shared starting point in (b) illustrates near‐saturated target binding before competitor addition; unchanged baseline splicing does not itself establish saturation. The curves contain no experimental data or assigned threshold. Binding and splicing are separate readouts and need not have the same slope.

The chosen increase in nuclear TDP‐43 should leave both cryptic exon inclusion and constitutive exon skipping unchanged in the control‐oligonucleotide and mock‐transfection conditions. Doses that independently disrupt splicing should be excluded (Carmen‐Orozco et al. 2024). Changes in nuclear localization or assembly must also be recorded. Because (GU)_16_ induces high‐molecular‐weight TDP‐43‐containing complexes, target displacement and protein rescue would not alone distinguish binding‐site competition from assembly‐mediated restriction of target access (Zhang et al. 2024). The experiment could also be extended to human induced pluripotent stem cell (iPSC)‐derived neurons, using *UNC13A* cryptic exon inclusion as the readout (Brown et al. 2022; Ma et al. 2022). Parallel UPF1 knockdown can reveal cryptic transcripts otherwise lost to nonsense‐mediated decay (Brown et al. 2022).

### 5.2 RNA Abundance and HuR Binding Sites

The Sindbis virus 3′UTR provides a defined RNA competitor for HuR. In human 293T cells, transfection of this RNA shortened the half‐lives of endogenous *DDX58, TUT1, LEPROTL1*, and *RSPRY1* transcripts (Barnhart et al. 2013). The capped, polyadenylated 3′UTR RNA can be titrated alongside GemA60 control RNA. To test which region mediates the effect, separate fragments can be compared: one containing the three repeat sequence elements, and another containing the U‐rich element and conserved sequence element that carry high‐affinity HuR‐binding sites (Barnhart et al. 2013). Comparisons should use matched measured cytoplasmic RNA abundance. Formaldehyde‐stabilized HuR immunoprecipitation followed by RT‐qPCR can measure association with the competitor and the endogenous targets. Target half‐lives can be determined by an actinomycin D chase, with *KAT5* and *RNASEL* included as negative control transcripts. HuR immunofluorescence and nuclear–cytoplasmic fractionation should accompany these assays, including during the actinomycin D chase. Endogenous HuR protein abundance and *ELAVL1* poly(A)‐site usage should also be measured across the titration, since Sindbis infection shifts *ELAVL1* towards shorter, more translatable isoforms (Barnhart et al. 2013). This response cannot be assumed for the transfected 3′UTR alone. Repeating the RNA titration after a modest increase in HuR tests whether additional protein preserves target association and stability at a higher competitor burden. Binding‐site dependence and rescue at comparable competitor abundance would support competition. This design tests how RNA abundance and binding‐site content affect other transcripts.

### 5.3 Stress Granules and Functional RBP Availability

Stress‐granule recruitment does not necessarily remove an RBP from functional RNA complexes. In human U2OS cells, Bley et al. (2015) suppressed visible granules by combined depletion of G3BP1, TIA1, and TIAR during sodium arsenate treatment. IGF2BP1 still stabilized its endogenous targets *MYC*, *ACTB*, and *MAPK4*. Bley et al. also found that IGF2BP1 depletion reduced target transcript abundance similarly with and without visible granules.

A quantitative extension would ask whether the two conditions differ in how far IGF2BP1 can decline before target binding and stability begin to deteriorate. A graded IGF2BP1 knockdown can be compared between control cells and cells depleted of the three assembly factors. Target association can be measured by cross‐linked IGF2BP1 immunoprecipitation and RT‐qPCR, alongside actinomycin D measurements of target decay. *PPIA* provides the non‐target control. Imaging should quantify granules and the distribution of IGF2BP1, while immunoblotting and a translation assay check protein abundance, eIF2α phosphorylation, and translation inhibition. Greater loss of target binding and stability at the same IGF2BP1 abundance in granule‐forming cells would support reduced functional availability. Overlapping responses would argue against that interpretation. Because the depleted assembly factors also act outside granules, a difference between conditions would require further mechanistic testing before being attributed specifically to sequestration.

### 5.4 Autoregulatory Responses to RNA Competition

Whether increased RNA demand activates TDP‐43 autoregulation can be tested with GFP or luciferase reporters carrying either an intact *TARDBP* 3′ regulatory region or its ΔTDPBR deletion, which removes the TDP‐43‐binding region (Avendano‐Vazquez et al. 2012; Ayala et al. 2011). The assay is first checked by supplying additional TDP‐43. GFP or luciferase expression should decrease more strongly from the intact reporter than from the deletion reporter. This establishes the regulatory response before testing RNA competition.

The reporters can then be challenged with the (GU)_16_ and control oligonucleotides described in Section 5.1. Reporter RNA abundance and GFP fluorescence or luciferase activity should be measured over time, with each reporter compared with its own control condition. A selective increase from the intact reporter would support relief of repression through the *TARDBP* regulatory region when competing RNA binds TDP‐43. Reporter RNA adds TDP‐43‐binding sites, so its baseline abundance should be kept low enough to avoid perturbing endogenous TDP‐43 abundance or splicing.

The autoregulatory and cryptic‐exon reporter assays should first be validated in the same cell background. Functional recovery can then be followed in parallel cultures using the bichromatic *Adnp2* reporter, in which cryptic exon inclusion increases EGFP expression (Zhang et al. 2024). Reporter fluorescence and RT‐PCR of its splice products can be paired with RT‐qPCR of endogenous *TARDBP* mRNA and immunoblotting of whole‐cell and nuclear full‐length TDP‐43. Measuring both pools would help distinguish protein accumulation from altered nuclear partitioning. Recovery of splicing as TDP‐43 rises, while oligonucleotide abundance and TDP‐43 engagement persist, would be consistent with compensation. Because endogenous *TARDBP* feedback remains intact in both reporter conditions, this experiment cannot establish whether autoregulation is necessary for splicing recovery.

The first two designs test whether adding a defined RNA population redistributes an RBP and changes regulation of other transcripts. The latter two examine whether assembly or autoregulatory responses modify functional protein availability. Reduced target association followed by impaired regulation, dependence on the competitor's binding region, and rescue by additional protein would together support competition. Preserved regulation despite reduced association would identify a buffered range for the particular target. A regulatory defect without a detectable change in target association would not support displacement at the assay's resolution; altered binding at a critical site or time point could still be missed. These comparisons test the framework in defined systems without assuming that every change in RNA regulation reflects limiting RBP supply.

## 6 Conclusion

RNA–protein reciprocity links the sites presented by RNA to the protein available to regulate them. When one RNA population binds more of a shared RBP, other targets may lose access to it. Their response depends on how strongly they bind the protein and how much binding their regulation requires. This provides a biological basis for stable regulation over one range of conditions and greater sensitivity over another. The framework helps explain why similar RBP perturbations can have different consequences across targets and compartments. It also accommodates feedback and RNA‐dependent assembly, both of which can change protein availability. These relationships act alongside RNA structure, partner proteins, and other established determinants of RBP function. The examples reviewed here establish that RNA‐dependent redistribution can preserve regulation or contribute to its disruption. The next task is to determine how often it matters and how large its effects are. Experiments that alter a competing RNA while measuring local RBP binding and target regulation provide a direct route to answering those questions. They may also identify conditions in which restoring usable protein or reducing competing demand can recover regulation. Such interventions will require dose control because correcting a shortage and producing protein excess can have different consequences.

## Author Contributions

**Epaminondas Doxakis:** conceptualization, visualization, writing – original draft, investigation, writing – review and editing, project administration, funding acquisition, resources, formal analysis, methodology.

## Funding

This research was funded by the Greek General Secretariat for Research and Innovation (GSRI, TAEDR‐0535850, acronym BrainPrecision). The funder had no role in the conceptual development or preparation of the manuscript.

## Conflicts of Interest

The author declares no conflicts of interest.

## Data Availability

No new datasets were generated for this article.
